# The diagnosis of sepsis revisited - a challenge for young medical scientists in the 21st century

**DOI:** 10.1186/1754-9493-8-1

**Published:** 2014-01-02

**Authors:** Lawrence A Lynn

**Affiliations:** 1 The Sleep and Breathing Research Institute, 1275 Olentangy RR, Columbus 43212, Ohio, USA

## Abstract

In 1991, a well-meaning consensus group of thought leaders derived a simple definition for sepsis which required the breach of only a few static thresholds. More than 20 years later, this simple definition has calcified to become the gold standard for sepsis protocols and research. Yet sepsis clearly comprises a complex, dynamic, and relational distortion of human life. Given the profound scope of the loss of life worldwide, there is a need to disengage from the simple concepts of the past. There is an acute need to develop 21st century approaches which engage sepsis in its true form, as a complex, dynamic, and relational pattern of death.

## 

Those attending patient safety conferences this year are likely to hear that the incidence of unnecessary deaths due to medical errors in the US is equivalent to a jumbo jet airline crash occurring every day, and that the majority of these errors are caused by diagnostic delay. This airline analogy dates back 19 years to 1994 [1], providing us with another startling analogy. Since 1994 the number of lives lost in the US due to medical error is equivalent to the total loss of soldiers and civilians on both sides during World War I. In that horrific conflict the thought leaders (Generals) continued to send their courageous soldiers over the top without acknowledging that their tactics were outdated. Now, nearly a century later history repeats itself as medical “thought leaders” today attempt to rally demoralized health care workers in patient safety conferences, only to send them back into the trenches armed with an antiquated, simplistically incapable “diagnostic science” from the past century.

Certainly, bedside sepsis diagnostics has made little progress since the late 1980s. In some respects, arguably the diagnostic definition of sepsis has actually declined. This isn’t surprising since much of the unnecessary loss of life from sepsis highlighted over the past decade has been due to its delayed detection. To understand why this is happening, we have only to review the recent history of sepsis diagnostics.

In the 1970s and early 80s there was a rapid growth in the understanding of the complex patho-physiologic elements comprising sepsis. In those days physicians considered sepsis to be a complex dynamic relational pattern of symptoms and laboratory findings, and concluded that sepsis was present when the patient exhibited a combination of symptoms and laboratory findings consistent with sepsis. This was our traditional “expert” method of medical diagnostics. The problem with this method is that it was complex and difficult to both reproduce and quantify. Then with the 1980s, there came a strong push to develop solid “scientific” methods of medical diagnostics, which would be simple enough to easily learn, copy accurately, and disseminate. The most popular of these emerging methods at that time was based on a new theory of medical diagnostics called “threshold decision making” [2].

Thresholds are now so much a part of 21^st^ century medical diagnostics that it might surprise those who trained in the past 20 years to learn that formalized threshold decision making is quite new. The threshold theory driving this approach is a reductionist (simplification based) theory that conceptualizes human disease as being best defined by one or more thresholds often derived from laboratory or vital sign testing. Threshold decision making holds that the clinician can either mathematically or mentally combine pretest probabilities of conditions with the sensitivity and specificity of one or more breached thresholds to determine the overall probability of an acute disease at the bedside.

Regarding sepsis, a typical question an adroit research clinician might ask while applying threshold decision making is, “What might be the expected sensitivity and specificity for sepsis when using an elevated biomarker threshold value in an adult patient presenting to the ER with symptoms suggesting an infection?” The same clinician might further ask whether this sensitivity and specificity is better than that from a WBC count in the same patient population. He or she might then prospectively sample venous blood in a very large population of randomized patients and apply a standard sepsis definition to determine which patients had sepsis and which did not. The next step would be to compare different threshold levels of biomarker in those patients meeting the definition of sepsis and in those patients who did not in order to identify the cutoff value for the biomarker with the best sensitivity and specificity for sepsis. The sensitivity and specificity of this biomarker’s threshold value could then be compared with optimal threshold values for WBC counts to determine which test is more reliable.

This may at first blush appear to be logical and scientifically sound, but a dose of skepticism is called for. One reason for doubting this methodology is that threshold decision making, when applied to sepsis diagnostics, hasn’t worked very well. Hearing the same jumbo jet airliner crash analogy repeated for 19 years straight should be an obvious clue, and certainly if any science based on a new theory is applied for 2 decades with persistently poor performances to show for it, the fundamental theory should be reconsidered.

Why is threshold decision making theory unsound regarding sepsis? One problem is that it requires the separation of a population into two distinct groups. Clinical “theorists” must first have an accurate, reliable definition for the disease, in this case sepsis, and then group the population into a “disease group” and “no disease group”. Unfortunately, in the 1980s there was no reliable definition of sepsis to allow for this appropriate separation. But this did not matter because threshold decision making requires research clinicians to make this reductionist move regardless, even when a true definition of disease does not exist. Later, as we will show, research clinicians did decide to keep form by using their best consensus guess at defining sepsis first, which then would serve to separate the groups as described above. However, in the early 1980s it was up to each research group to choose their own definition.

Obviously, whenever a range of “best guess” definitions of disease are used in clinical research to separate populations, the results of that research become subjective and unreliable. One minor error in the guessed definition can render inaccurate results. Furthermore, since each individual research group was allowed to guess their own definition of sepsis for each of their own clinical trials, then it’s reasonable to expect each guess to be a little different. By the late 1980s the thought leaders of this diagnostic science realized they had a major problem on their hands. Researchers were applying threshold theory to multiple clinical trials on sepsis, yet each trial used different definitions for sepsis, likening any study comparisons to the proverbial apples to oranges. Both the validity of this research and its applicability to the bedside management of septic patients were never clear. The leaders had to find a solution, and because the problem of excessive deaths due to sepsis was particularly acute, the solution had to be found quickly.

One available option would have been to perform a multicenter clinical trial in patients with severe infection with bacteremia resulting in death or near death, and then select the sepsis definition as a function of the findings from this trial. However, mathematical reductionism had become increasing popular in 1980s medical diagnostics, and most scientists had not yet perceived the weaknesses of reductionist threshold theory and the dangerous anomalies it commonly induces. About this same time a new validation methodology called “consensus theory” began to emerge. This theory held that reliable, valid scientific judgment could be rendered through a formal process of expert consensus derived from a heterogeneous population of experts. This was popularized by the Delphi method of decision making. Consensus theory offered the quickest solution for the need of a “legitimate” sepsis definition, and thought leaders jumped at the opportunity to take this new approach. This application of consensus theory would be touted to allow for transparency and standardization across all clinical care regarding sepsis, as well as all related clinical trials that involved this disease. Unfortunately, history has shown us that these conclusions were erroneous, as was the expert consensus definition derived for sepsis itself. Alas, the weakness of expert consensus, pointed out so graphically by Thomas Kuhn in the 1960s, had been largely forgotten in the heady days of late 20^th^ century medical discovery. In fairness, formal threshold decision making was only a little over 10 years old at the time, and its weaknesses were not yet well understood. Indeed, the merging of the threshold theory with consensus theory seemed quite reasonable during those years. The well-meant choice to use a hybridization of two new theories of decision processing was the unfortunate beginning that led to the present state of sepsis diagnostics, which I discuss below.

With the advantage of hind sight and robust analytics, we can readily look back and identify the mistakes made by the original sepsis consensus group in 1991 [3], which then repeated in 2001 [4], once again repeating in 2012 [5]. The most evident mistake made by this group was to define sepsis in terms of a few simple static thresholds. Sepsis is known today to be a very complex, dynamic and relational distortion of human life. It cannot be reasonably, accurately, or reliably defined with a few threshold values, nor can it be defined without time factored into the definition. Perhaps the original thought leaders suspected this all along and were simply trying to start the process of developing a robust definition in 1991. However, for reasons not entirely clear, this original definition calcified, becoming firmly ensconced as our gold standard [5], despite its mediocre performance in terms of mortality and morbidity.

Let us take a deeper look at how simple expert guessing can evolve over 20 years to become a gold standard. Scientists, despite their reputation for being independent thinkers, have always been prone to follow those they perceive as legitimate “thought leaders.” Scientific consensus directives that are repetitively delivered from central authority exhibit a “Fabian” quality. While actual scientific discoveries gain validity through repetitive experimental confirmation, consensus based “discoveries” gain their “validity” by proclamation and repetition. To this point, in 2001 our thought leaders wishfully argued that the definition of sepsis, which they had been forced to guess as a function of the perceived need for consensus in 1991, was “robust” because it had been “used” (in contrast to tested or proven) in clinical trials.

Certainly in the early 1990s, the newly published sepsis thresholds were recognized for what they actually were, consensus derived guesses. However, over time these guesses grew in stature, gaining prominence as they were increasing cited in formal consensus statements and used as gold standards in clinical trials. Today, our thought leaders no longer see the need to argue for their “validity” as components of the definition of sepsis, but simply assume this validity, and quote the old sepsis definition in new consensus guidelines [5], as one might quote a physics formula for torque.

With this understanding of today’s definition of sepsis, let us look a little deeper into its actual components. The first thing to notice is that the definition is actually a simple summation of a plurality of static thresholds where just two threshold breaches (along with the perceived state of infection) are all that is required to complete the criteria for a positive diagnosis. In today’s vernacular (and in the software database tools being offered to physicians today), sepsis has become a “three click” diagnosis. In an example of just how simple the diagnosis of sepsis is within the standard definition, a fever of 100.7 and a heart rate of 95 will render a diagnosis of “sepsis”. Most of us met this simple definition the last time we had a mild case of self-limiting influenza.

Additional evidence of oversimplification is provided by the lack of temporal relationships of the threshold values in the sepsis definition. One needs no mathematical training to understand that a time critical process like sepsis should include at least one aspect of time. Regrettably the concept that this complex patho-physiologic, time-sensitive process can be quantified by a simple occurrence of two static threshold values continues to remain the standard for sepsis protocols and research.

As another example of problems arising from the use of static thresholds in defining sepsis, it is actually possible today for a patient to initially meet the standard definition of sepsis, and then even as this sepsis progresses to a more severe state over time, to no longer “have it” according to the threshold criteria. The WBC count often rises to high levels and then falls as neutrophils are expended while sepsis progresses. As a WBC count falls from its initial peak, it actually may cross back into its “normal” range. Blood samples taken at that specific point in time will exhibit a completely normal WBC count so that, should the patient’s sepsis diagnosis have been initially defined using the WBC count as one of the two threshold components needed, the sepsis definition is now no longer met. Obviously this particular patient hasn’t been miraculously cured, and will more than likely continue to be aggressively managed, but this does not necessarily bode well for those patients being initially tested in this more advanced time-sensitive septic conundrum. The consensus group actually recognized this problem, but rather than challenging the validity of its static threshold driven definition, attempted to solve the problem by adding yet another static threshold of 10% immature neutrophils.

One important cause for consensus science related error can be associated with the homogeneity of its thought leaders’ training. For any consensus method to be effective, a sufficient number of the scientists involved should have different training backgrounds. Otherwise, the consensus tends to simply reflect the training and bias of their common backgrounds. Another interesting bias issue that is pertinent here because of the heavy reliance on numeric thresholds, comes from the possibility that expert groups may subconsciously skew these threshold values toward certain numbers because of social pressures from other scientific disciplines. As noted earlier, this appears to have occurred in the selection of a threshold value for immature WBC counts, where our experts selected a cutoff value of 10. This same group also selected the threshold cutoff of 100 for the number of platelets, and the cutoff of 100-10 for the threshold of heart rate and the same 100-10 for the threshold of systolic blood pressure. This affinity for the numbers 10 and 100 (which have no inherent patho-physiologic significance) may have, in part, been driven by their stated desire to “keep the definition simple”, as well as the “go metric” initiatives of the late 20^th^ century when most of these leaders were likely influenced during their high school and undergraduate training.

It should be clear now that our current standard definition for sepsis is both incomplete and flawed. For this reason, clinical trials that rely on this standard sepsis definition are equally flawed and unreliable. Yet a more far reaching problem may be that the clinicians using these simple static definitions for the last 20 years have no other way to think of this dynamic condition except in terms of flawed static thresholds that often condemn patients to misdiagnosis and diagnostic delay. One might perceive it mitigating that the 20^th^ century experts selected thresholds that were very sensitive, but we now know that this well-meaning approach to the selection of an “oversensitive” (nonspecific) definition for a potentially fatal disease results in alarm fatigue and diagnostic delay due to the “crying wolf” phenomena from excessive false positive warnings [6,7]. Young physicians, having trusted in the “scientific” basis of these simple decision making methods, are now inclined to anchor on only those thresholds that can be rendered intentionally more specific (fewer false positives) by increasing their threshold cutoff values. This does reduce the risk for alarm fatigue, but unfortunately these rendered threshold breaches occur only later in the septic process, once again inherently creating diagnostic delay [8].

One residual argument for the use of the simple sepsis definition is that it is easy to learn. It allows complex cases of sepsis to be detected by focusing on simple criteria, which most any healthcare worker can reliably remember. This argument was made by the sepsis definition consensus group in 1991 and again in 2001 [3,4]. Its rationale was consistent with another prevailing influence of late 20^th^ century, the “law” of parsimony (Occam’s razor) which was often misinterpreted as indicating that a smaller number of variables is preferable to more. The perceived value of parsimony was enhanced by the application of multiple linear regressions, neural nets, genetic algorithms, swarm intelligence and other analytic methods of the 20^th^ century. The high hopes many scientists had in the late 20^th^ century that these artificial intelligence methods would be able to provide bedside diagnoses for individual patients was mitigated by the recognition that these statistical methods were vulnerable to greater error in the presence of greater numbers of variables. Unfortunately, the response went too far and, by using just a two simple threshold values, they rendered a definition of sepsis which was nonspecific, and prone to unacceptably high numbers of false positives [6]. This is an expected collateral effect whenever excessive emphasis on parsimony prevails. The proper application of parsimony in science was perhaps best characterized by Albert Einstein when he said, “Everything should be made as simple as possible, but not one bit simpler”.

Another more pragmatic argument for the present consensus definition is that without a unified definition to fall back on, the basis for diagnosis is not measurable, and therefore cannot comprise a basis for evidence-based medicine, which requires the outcome effect be relatable to a measurable decision to treat. This widely disseminated, well-meant argument is no longer intellectually tenable. There is no good reason why the definition of sepsis needs to be what it is today. There is no good reason why the definition of sepsis cannot include the factor of time or why it needs to be comprised of arbitrary thresholds that are simply the best 20 year old guesses of experts from another era, and not the findings from robust clinical trials.

Given that the present definition of sepsis is simply a 20 year old guess, what might the actual definition of sepsis be? Is it possible to accommodate the dynamic relational complexity of this disorder and still have a diagnostic definition for sepsis that is simple enough to use at the bedside? To answer this question, it is reasonable to look at other disciplines that have escaped their own simplistic constructs. In the early 1900s, the construct of the universe was static and much simpler than it really was. Like the sepsis scientists of today, Edwin Hubble was certainly influenced by his mentors’ dogma, but as an excellent scientist and student of the history of science, he was always ready to accept, with proof, that these standard concepts were incomplete and possibly wrong. When Dr. Hubble gazed out into the static universe of his mentors he opened his mind as well as his eyes, perceiving a dynamic, expanding universe, comprised of billions of distant dynamic galaxies rather than individual distant stars and nebulae. Each of these galaxies and their rotational masses contained a central black hole comprising dynamic distortions in space and time that were never previously part of its fundamental construct. Even now, building on these discoveries and new tools to see into the complexity of space, the constructs of the universe are radically different from those existing only 30 years ago. Our present knowledge of the universe was only possible because a new group of scientists were ready to accept that even the best contemporary science and its most intelligent thought leaders can eventually find themselves on the wrong path. In fact this has proven inevitable. It is the one constant remaining in man’s noble quest for scientifically proven knowledge.

Sepsis considered without the constraints of past dogma, might be seen, not as defined by a plurality of macro-thresholds, but rather as a dynamic relational distortion of the fabric of the time matrix of biologic particle densities. In health, biologic particle densities (e.g. neutrophil count, platelet count, the concentration of ions, the partial pressures of gasses, and molecules such as albumin) are maintained within tight rational variances comprising a stable time matrix. Upon the development of infection, this time matrix of biologic particle densities becomes distorted. The initial distortion and the progression of the infection further pulls on, and distorts other parts of the matrix over time generating a complex, cascading, dynamic relational pattern of distortion eventually to involve the entire matrix if left unchecked. In the future, definitions of sepsis may be comprised of time sensitive motion images of these distortions [8].

However, the purpose of this letter is not to define an improved diagnostic definition for sepsis. Rather, I hope to encourage young clinicians, researchers, and scientists to arouse to the acute need for better methods and models to define our currently incompetent diagnostic definition. Together, we must accept that we have clung to present definition of sepsis, which has been repetitively proclaimed in 1992, 2002, and finally in the 2012 consensus conference statements, only as a function of its age and the pedigree and respect we hold for its advocates. Regrettably, like the emperor that has no clothes, this standard sepsis definition has be seen for what it really is, simply a 20 year old embellished, but heavily flawed guess. Research performed with this definition will consistently render variable and unreliable results. Worse, the results of clinical trials using this simple definition have the potential to inflate the diagnosis of sepsis and thereby generate an inflated (false) sense of improved outcomes due to its intrinsic diagnostic flaws. For example, by including patients with mild infection in the diagnosis of sepsis, the number of survivors will increase and this may be readily (but incorrectly) attributed to the intervention applied in the protocol which used this definition.

However, many young scientists trained under the paternal, central control of consensus science (which directs government grants and influences publications), may be more inclined to simply call for another consensus meeting of current “thought leaders” to select “better” thresholds [7], rather than challenge the fundamental flaws of the current methodology. This has been the repetitive pattern identified by Dr. Thomas Kuhn from his thought provoking study of centuries of past scientific revolutions. As Dr. Kuhn has pointed out “…science students accept theories on the authority of teacher and text, not because of evidence" [9].

As clinicians, when a young patient is not improving after we have applied all the treatment indicated based on our assessment, we are taught to start the diagnostic process over, at the very beginning, as if everything we initially concluded was incorrect. It is time for our sepsis thought leaders to set aside bias and apply this same degree of diligence. Unfortunately, Dr. Kuhn teaches that regardless of the anomalies induce by their consensus, the thought leaders will not be able to abandon their dogma. “Though they may begin to lose faith and then to consider alternatives, they do not renounce the paradigm that has led them into crisis” [9]. Therefore, it falls to today’s young clinicians, researchers, and scientists to take the lead ASAP in the field of sepsis diagnostic science to stop these “silent” daily jumbo jet airline crashes.

Lawrence A. Lynn, DO, FCCP.

## Competing interest

Dr. Lynn has issued and pending patents which relate to the detection of sepsis and other clinical conditions by imaging time-matrix distortions. He is the owner of Lyntek Medical Technologies which develops sepsis detection software. He is the co-inventor of PateintStormTracker, visualization software for imaging and presentation of time-matrix distortions. He has licensed patents for monitor based pattern recognition to Covidien.
